# Identification of neural networks preferentially engaged by epileptogenic mass lesions through lesion network mapping analysis

**DOI:** 10.1038/s41598-020-67626-x

**Published:** 2020-07-03

**Authors:** Alireza M. Mansouri, Jürgen Germann, Alexandre Boutet, Gavin J. B. Elias, Karim Mithani, Clement T. Chow, Brij Karmur, George M. Ibrahim, Mary Pat McAndrews, Andres M. Lozano, Gelareh Zadeh, Taufik A. Valiante

**Affiliations:** 1 Department of Neurosurgery, Penn State Health, Hershey, PA USA; 2 University Health Network, Toronto, ON USA; 3 Joint Department of Medical Imaging, University of Toronto, Toronto, ON USA; 4 Faculty of Medicine, University of Toronto, Toronto, ON USA; 5 Program in Neuroscience and Mental Health, Sickkids Research Institute, Toronto, ON USA; 6 Division of Neurosurgery, The Hospital for Sick Children, Toronto, ON USA; 7 Department of Surgery, Institute of Biomaterials and Biomedical Engineering, University of Toronto, Toronto, ON USA; 8 Department of Neuropsychology, University Health Network, Toronto, ON USA; 9 Division of Neurosurgery, University Health Network, Toronto, ON USA

**Keywords:** Diseases of the nervous system, Neural circuits

## Abstract

Lesion network mapping (LNM) has been applied to *true* lesions (e.g., cerebrovascular lesions in stroke) to identify functionally connected brain networks. No previous studies have utilized LNM for analysis of intra-axial mass lesions. Here, we implemented LNM for identification of potentially vulnerable epileptogenic networks in mass lesions causing medically-refractory epilepsy (MRE). Intra-axial brain lesions were manually segmented in patients with MRE seen at our institution (EL_INST). These lesions were then normalized to standard space and used as seeds in a high-resolution normative resting state functional magnetic resonance imaging template. The resulting connectivity maps were first thresholded (*p*_*Bonferroni_cor*_ < 0.05) and binarized; the thresholded binarized connectivity maps were subsequently summed to produce overall group connectivity maps, which were compared with established resting-state networks to identify potential networks prone to epileptogenicity. To validate our data, this approach was also applied to an external dataset of epileptogenic lesions identified from the literature (EL_LIT). As an additional exploratory analysis, we also segmented and computed the connectivity of institutional non-epileptogenic lesions (NEL_INST), calculating voxel-wise odds ratios (VORs) to identify voxels more likely to be functionally-connected with EL_INST versus NEL_INST. To ensure connectivity results were not driven by anatomical overlap, the extent of lesion overlap between EL_INST, and EL_LIT and NEL_INST was assessed using the Dice Similarity Coefficient (DSC, lower index ~ less overlap). Twenty-eight patients from our institution were included (EL_INST: 17 patients, 17 lesions, 10 low-grade glioma, 3 cavernoma, 4 focal cortical dysplasia; NEL_INST: 11 patients, 33 lesions, all brain metastases). An additional 23 cases (25 lesions) with similar characteristics to the EL_INST data were identified from the literature (EL_LIT). Despite minimal anatomical overlap of lesions, both EL_INST and EL_LIT showed greatest functional connectivity overlap with structures in the Default Mode Network, Frontoparietal Network, Ventral Attention Network, and the Limbic Network—with percentage volume overlap of 19.5%, 19.1%, 19.1%, and 12.5%, respectively—suggesting them as networks consistently engaged by epileptogenic mass lesions. Our exploratory analysis moreover showed that the mesial frontal lobes, parahippocampal gyrus, and lateral temporal neocortex were at least twice as likely to be functionally connected with the EL_INST compared to the NEL_INST group (i.e. Peak VOR > 2.0); canonical resting-state networks preferentially engaged by EL_INSTs were the Limbic and the Frontoparietal Networks (Mean VOR > 1.5). In this proof of concept study, we demonstrate the feasibility of LNM for intra-axial mass lesions by showing that ELs have discrete functional connections and may preferentially engage in discrete resting-state networks. Thus, the underlying normative neural circuitry may, in part, explain the propensity of particular lesions toward the development of MRE. If prospectively validated, this has ramifications for patient counseling along with both approach and timing of surgery for lesions in locations prone to development of MRE.

## Introduction

While medically refractory epilepsy (MRE) in adults is most commonly diagnosed in mesial temporal lobe epilepsy (mTLE), it is not uncommon in individuals with intra-axial brain lesions such as low-grade gliomas (LGGs, up to 50%), cavernomas (up to 40%), and focal cortical dysplasia (FCD)^1–3^. The pathophysiology of seizures and epilepsy in these individuals is multi-factorial. While cortical lesions are prone to epilepsy, other more complex factors related to the lesion microenvironment, such as peri-lesional inflammatory changes, imbalance of excitatory and inhibitory neurotransmitters, and metabolic changes, have been implicated^4–7^. Furthermore, gene expression profiling of glioneuronal tumors and some FCDs have identified common pathways in both pathologies that contribute to pathogenesis and epileptogenesis^8^. Broader changes related to whole-brain connectivity may also play an important role by facilitating an altered threshold for seizures^9^. Several lines of investigation have supported the notion of epilepsy, whether focal or generalized, as a large-scale, network-wide disorder, rather than one isolated to particular region(s) of the brain^10–13^. This, in part, explains the cognitive and psychological manifestations in individuals with focal epilepsy^14^.

At present, it remains unclear why identical lesions in different brain regions have differing propensity to induce seizures. One putative hypothesis relates to the underlying neural circuitry they are more likely to engage. Several neural networks have been uniquely implicated in epileptogenicity in both humans and non-human primates^15–17^. An emerging approach to understand the relation between intra-axial brain lesions causing epilepsy and the underlying neural circuitry involves leveraging high-quality resting state functional magnetic resonance imaging (rsfMRI) data from large samples of healthy subjects (i.e., normative data). This facilitates explorations of network connectivity in subjects of interest who lack native functional neuroimaging in routine clinical MRI protocols—a common scenario in the routine clinical practice involving people with brain tumors—but also informs the relation between lesions in an individual patient and normative brain connectivity^18,19^. This approach, known as lesion network mapping (LNM), has been applied in numerous recent studies assessing clinical symptoms/syndromes attributable to focal lesions in diverse anatomical brain locations^20–22^. LNM allows for a better understanding of a distinct neurological manifestation—such as seizures—originating from lesions in anatomically disparate locations. Extending the use of this method to epileptogenic tumors, LNM analysis has the potential to identify canonical resting-state networks that are vulnerable to epileptogenesis secondary to an offending lesion, which may not be necessarily explainable by simple anatomy. Recently, LNM was used to postulate a connectomic rationale for heterogeneous seizure outcomes following MR-guided laser interstitial thermal therapy for epilepsy^23,24^. In addition, a similar approach was employed to identify networks that may contribute to deep brain stimulation-induced seizures^17,25^. To date, however, LNM analysis has not been applied for larger intra-axial mass lesions causing epilepsy.

In this proof of concept study, we applied the LNM method to a comparison of our center’s epileptogenic lesions (EL_INST) with non-epileptogenic lesions (NEL_INST). To accomplish this, individuals harboring tumors that caused epilepsy were identified and compared with lesions in other individuals who did not have seizures as part of their clinical presentation. The LNM method, using the patients’ own structural imaging superimposed on normative rsfMRI atlas data, was then used to identify resting-state networks portending vulnerability to epileptogenicity. For comparative purposes, the same LNM approach was applied to non-epileptogenic mass lesions derived from a second patient cohort at our institution (NEL_INST) and to epileptogenic mass lesions sourced from the published literature (EL_LIT). We hypothesized that (1) EL would have distinct connectivity patterns; (2) this pattern should be externally valid with respect to epileptogenic lesions encountered outside our institution and would involve resting-state networks known to be implicated in epileptogenicity; and (3) the functional connectivity pattern associated with ELs would differ from that associated with NELs.

Through demonstration of the feasibility of LNM for mass lesions, new hypotheses regarding connectivity patterns implicated in epileptogenicity can be tested. Furthermore, once verified, this method could be applied toward patient counseling and surgical planning, as earlier surgery could be applied for lesions in locations thought to be more prone to development of MRE based on their brain-wide connectivity profile. This is especially valuable because not all patients, particularly those with neoplastic lesions, undergo native functional imaging in a routine clinical scenario.

## Methods

### Institutional cohort case selection

This was a retrospective study conducted at the University Health Network following institutional research ethics board approval (Neurosciences committee, study ID UHN13-6399). All research was performed in accordance with relevant guidelines/regulations. Informed consent was waived by institutional research ethics board at the University Health Network due to the retrospective nature of the study.

We reviewed clinical and imaging data of individuals with intra-axial brain tumors/lesions resulting in MRE (i.e., the EL_INST cohort) and without prior surgical resective procedures who were identified as possible surgical candidates (January 2007–October 2018). Comprising the NEL_INST cohort (control population) were seizure-free individuals with brain tumors with no prior cranial surgical procedures (October 2018). Only cases with a comprehensively documented clinical exam that specifically detailed the presence or absence of a diagnosis of epilepsy, and corresponding high resolution (3D sequences with isotropic voxels ≤ 2 mm) T1-weighted (T1W) structural images (SPGR or MPRAGE) were included. For both non-contrast T1-weighted and post-contrast T1-weighted images, the acquisition parameters were TR = 7.5–9.0 ms, TE = 3.0–4.2, flip angle = 12–15°, isotropic voxel ≤ 2 × 2 × 2 mm. Among patients in the EL_INST cohort, data pertaining to seizure-freedom status at 12 months following surgery was also extracted.

### Systematic review of the literature to identify ELs for external validation

In order to externally validate functional connectivity patterns potentially implicated in epileptogenicity (according to analysis of our EL_INST cohort), additional intra-axial tumors causing MRE were identified from the literature. This approach has been successfully undertaken in several prior similar studies^20–22,26^. A systematic search for published reports of ELs histologically comparable to our own cohort was conducted on March 27, 2020 using the MEDLINE database, using medical subject headings (MeSH) and free-text terms related to “magnetic resonance imaging” and “low grade glioma (astrocytoma, diffuse glioma, ganglioglioma, and oligodendroglioma)” and “epilepsy”. A total of 320 articles were retrieved. Following screening by two reviewers (CTC, AB), 19 articles were deemed eligible for analysis. Any disagreements were settled by a consensus decision after discussion with the third reviewer (AM) to confirm presence of an intra-axial glioma with clear imaging and epilepsy attributed to the lesion. Study inclusion criteria comprised English language, focused on adults (age ≥ 18 years old), and presence of an MRI image of an intra-axial lesion presumed to cause epilepsy. Data extraction was performed independently by two authors (AM, CTC) using a pre-constructed spreadsheet (see Supplementary Table 1). Overall, 23 cases (25 lesions) were identified; these comprised the EL_LIT cohort and were used for external validation of the EL_INST cohort.

**Table 1:** Case summary of lesions used in lesion network mapping analysis.

	Epileptogenic lesions (institutional)	Non-epileptogenic lesions (institutional)	Epileptogenic lesions (literature)
N cases	17	11	23
N lesions	17	33	25
Sex (M/F/unknown)	7/10/0	6/5/0	14/8/1
Mean age, years (SD)	33.7 (11.8)	68.5 (8.4)	36.7 (12.3)
Median lesion volume (cc), range	3,450.5 (374–44,038)	737 (44–5,916)	N/A
Median Thresholded Connectivity Volume (cc), range	325.8 (74.1–590.6)	294.4 (78.8–630.9)	N/A
Pathology	Diffuse Grade II Glioma = 10 Cavernoma = 3 Cortical dysplasia = 4	Brain metastases = 11	Diffuse Grade II Glioma = 17 Hamartoma = 1 Neoplasm = 1 Ganglioglioma = 4
**Lesion location**^a^			
Frontal Temporal Parietal Occipital Insula Cerebellum Brainstem	4 12 1 0 0 0 0	9 8 8 3 1 3 1	14 12 2 1 3 0 0
**Lesion laterality**			
Left Right Midline	13 4 0	15 16 2	15 10 0

^a^For epileptogenic lesions (literature), the sum of lesion locations does not total 25 as some lesions were located in multiple lobes.

### Lesion segmentation

High resolution T1-weighted structural images were used to delineate intra-axial lesions for both institutional lesion cohorts (EL_INST and NEL_INST). Lesions were manually segmented in native space using MRIcron (https://www.nitrc.org/projects/mricron)^23^. Segmentation was performed by two independent authors (AM, AB) in the axial plane and confirmed for accuracy on the coronal and sagittal planes. While all segmentations were performed on non-contrast T1-weighted images, additional image sequences were referred to in order to ensure segmentation accuracy. For example, Fluid-Attenuated Inversion Recovery (FLAIR) sequences were used to guide segmentation of LGGs and FCDs, Gradient Resolution Echo (GRE) sequences to guide segmentation of cavernomas, and post-gadolinium T1-weighted contrast images to guide segmentation of metastatic lesions. Only the mass lesion itself was segmented; any surrounding edema was excluded.

The T1-weighted images were registered to the MNI152 template in a stepwise iterative process using linear (FLIRT) followed by nonlinear (FNIRT) registration techniques^25,27^. For each step, the previously segmented lesion mask was applied as an input-weighting volume^25^ to avoid registration distortion from abnormal voxel intensities within the lesion areas. Each transformation was then applied to the matching lesion mask, transferring it into MNI space for group-level analysis.

**Figure 1:**
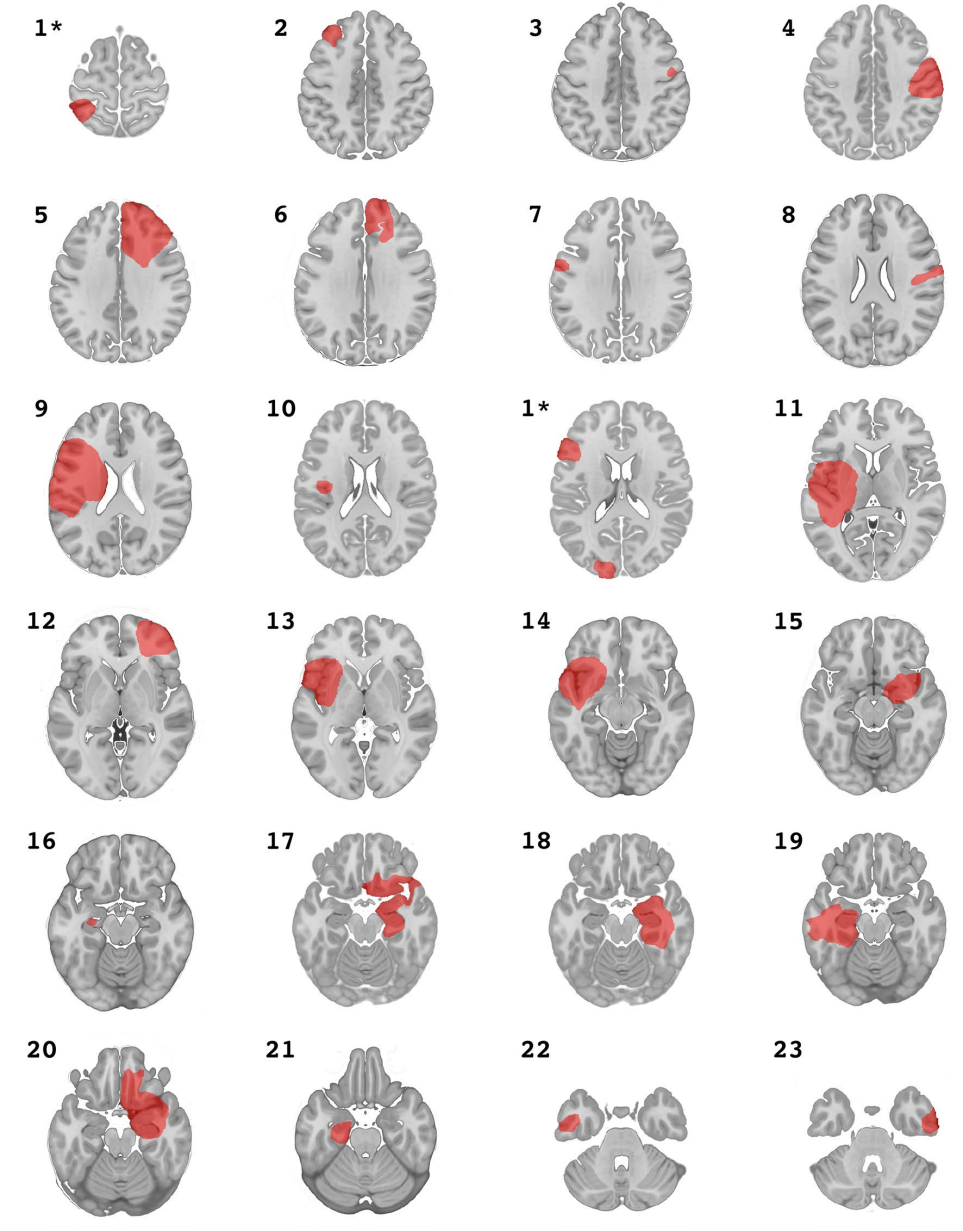
General location and appearance of lesions segmented from the literature.

For the EL_LIT cohort, segmentation of available two-dimensional tumor images was performed manually on a standard template brain (MNI152 asymmetric brain). This brain was resampled at 0.5 × 0.5 × 2 mm to preserve anatomical contrast in the axial plane and thereby better delineate/identify the lesion, similar to methods described previously^20–22,26^. All 25 lesions from the eligible published figures were accurately segmented—referencing neuroanatomical landmarks—onto the 2D axial plane of the template brain (Fig. 1).

### Spatial overlap across segmented lesions

The extent of physical overlap between (i) EL_INSTs and NEL_INSTs, and (ii) EL_INSTs and EL_LITs was examined using the Dice similarity coefficient (DSC, also known as the Sørensen-Dice coefficient or index), a metric that evaluates overlap between binarized volumes according to the following formula:

$$DSC = \frac{2|X \cap Y|}{{|X| + |T|}}$$

In this formula, |X| and |Y| are the cardinalities of the two sets (i.e. the number of elements in each set). The DSC equals twice the number of elements common to both sets divided by the sum of the number of elements in each set.

### Lesion network mapping

For both our in-house treated dataset (i.e., patients treated at UHN) and the literature-derived validation dataset, the segmented lesion masks were subsequently used as seeds (regions-of-interests) for the functional connectomic mapping analysis (in-house MATLAB script, The MathWorks, Inc., Version R2018a. Natick, MA, USA31-34). This analysis utilized a normative dataset compiled from rsfMRI scans of 1,000 healthy subjects, as reported previously^20,24,28^. Each subject was scanned once or twice (1.7 times per subject on average) with a 6.2 min-long echo-planar imaging sequence (124 time points; 3 × 3 × 3 voxel size, TR 3,000 ms, TE 30 ms, flip angle 85°) in order to acquire rsfMRI data.

Whole-brain connectivity r-maps were generated for each individual seed using the entire normative dataset. For each seed, a connectivity r-map describing the correlation between the seed and every voxel in the brain—on the basis of the averaged low-frequency blood-oxygen-level-dependent (BOLD) signal fluctuations sampled across the 1,000 normative subjects—was obtained. The r-map describes the pairwise correlation between the BOLD time courses of the seed and every voxel in the brain. For patients with multiple lesions, these were used collectively as a single seed for connectivity mapping. To define meaningful networks, each patient’s r-map was converted to a t-map with a known *p* value distribution; this t-map was then Bonferroni-corrected for multiple comparisons at t = 5.1 (*p*cor < 0.05) across the entire brain^19^. The thresholded t-maps were then binarized such that all subsequent analyses looked solely at the topographical relationship between the different groups’ significant (*p*_*cor*_ < 0.05) connectivity patterns, as opposed to considering subtle variations in how strongly a given region is connected to either group^24^. This conservative strategy addresses some of the limitations associated with using normative healthy patient data in epilepsy patients, whose brain functional connectivity may differ substantially from healthy controls^29^. Specifically, the use of stringent thresholding and binarizing was intended to avoid over-interpretation of more granular connectivity patterns that exist in the normative dataset employed here but are less likely to hold true in epilepsy patients.

Using the individual thresholded and binarized connectivity maps, summed connectivity maps were separately computed for the EL_INST and EL_LIT to obtain the overall connectivity patterns linked with each group. The higher the voxel value in each cohort’s summed image, the greater the number of individual connectivity maps that overlapped with that voxel. To also assess network-level engagement, overlap between the summed connectivity maps and 7 canonical resting state networks (https://surfer.nmr.mgh.harvard.edu/fswiki/CorticalParcellation_Yeo2011)^24,28^ was assessed.

### Connectivity patterns of EL_INST versus NEL_INST

To complement our description of epileptogenic networks in EL_INST, we also conducted an exploratory analysis to determine the odds of particular resting-state networks being involved in epileptogenicity as opposed to non-epileptogenicity. This was done in order to clarify whether the networks or regions found to be connected to epileptogenic lesions were in fact *specifically* related to epilepsy or not, and was accomplished by a comparison between EL_INST and NEL_INST connectivity. The lesions from EL_LIT were excluded from this analysis due to concerns regarding the limitations of combining 2D segmentation approaches with actual 3D techniques possible using our in-house cases.

First, the thresholded binarized connectivity maps were separately summed across the EL_INSTs and NEL_INSTs (i.e., summed connectivity maps) in order to obtain the overall connectivity patterns linked with each cohort. The higher the voxel value in each summed connectivity map, the greater the number of individual connectivity maps that overlapped with that voxel.

The network connectivity specifically associated with EL_INSTs was then investigated by computing voxel-wise odds-ratio (VOR) maps that contrasted the binarized connectivity masks from each cohort. Here, the NEL_INST cohort served as the control. Generation of the VOR map was based on previously described methods^30^.

$$VOR = \frac{{Ve\left( {Nne - Vne} \right)}}{{Vne\left( {Ne - Ve} \right)}}$$

where Ne = number of epileptogenic lesions; Nne = number of non-epileptogenic lesions; Ve = number of epileptogenic lesions overlapping a specific voxel; Vne = number of non-epileptogenic lesions overlapping a specific voxel. Ve and Nne were interchanged in the formula to calculate the NEL_INST VOR map.

In order to systematically interpret the results of the VOR analysis in the context of anatomical brain regions, we overlaid the VOR maps on the AAL atlas, which is a well-established repository of standard masks of individual brain regions (e.g., cerebellum). Although the AAL atlas is an excellent tool for obtaining an overview of the anatomical distribution of connectivity, anatomy does not necessarily reflect functional divisions^31^. The average VOR value in each AAL ROI was then computed and those with peak VOR > 2.0 were displayed; this threshold was chosen to only display regions with the highest VOR of connectivity. Next, in order to investigate the potential differences between EL_INST and NEL_INST in the context of wider brain networks, the VOR maps of associated functional connectivity hubs implicated in epileptogenicity were also overlaid on the standardized atlas of seven canonical resting-state networks (https://surfer.nmr.mgh.harvard.edu/fswiki/CorticalParcellation_Yeo2011)^24,28^. To calculate mean VOR values for the resting-state networks, the liberal binary masks were derived for each. The mean value within each mask was then calculated for both the EL_INST and NEL_INST VOR map. Resting state networks with mean VOR > 1.5 were displayed in order to show only regions with the highest likelihood of functional connectivity.

### Statistical analyses

Depending on the distribution of the data, mean or median values were calculated along with standard deviation or range, respectively. Similarly, t-test or Wilcoxon Rank Sum tests were used to assess for statistical significance. A *p* value < 0.05 was deemed significant. All analyses were performed with SPSS v22.0 (IBM Corp) or R (https://www.r-project.org/, version 3.4.4) and RMINC (https://github.com/Mouse-Imaging-Centre/RMINC) for the imaging analyses.

## Results

### Clinical and lesion characteristics

**Figure 2:**
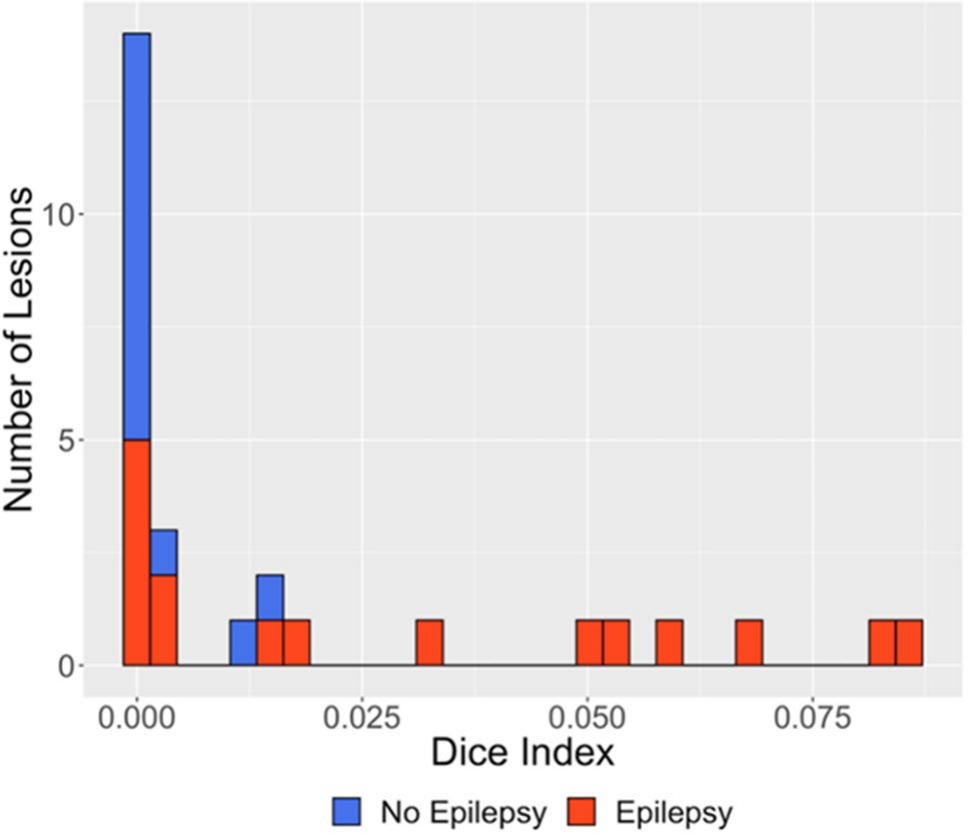
Dice similarity coefficient, quantifying degree of overlap between EL_INST and NEL_INSTs; smaller number indicates lower degree of overlap. Non-epileptogenic lesions = blue, epileptogenic lesions = red.

Based on our clinical and imaging inclusion criteria, 28 cases were included in this analysis (Table 1). The EL_INST cohort comprised 17 individuals each with a single intracranial lesion: LGGs were the most common pathology (11 of 17), followed by FCD (3 of 17) and cavernoma (3 of 17). The NEL_INST cohort comprised 11 individuals with a total of 33 intracranial metastatic lesions. On average, the EL_INST cohort was significantly younger than the NEL_INST cohort (Mean age ± SD: 33.7 ± 11.8 years versus 68.5 ± 8.4 years, *p* < 0.05). The majority of lesions in the EL_INST cohort were located in the temporal lobe (12 of 17) and lateralized to the left side (13 of 17); whereas, lesions in the NEL_INST cohort were relatively evenly distributed across the brain (Table 1). The DSC (which reflects extent of lesion overlap) for both groups was low overall, with EL_INST lesions demonstrating slightly greater lesion overlap within the cohort (Fig. 2). Following surgery for MRE, 15 of 17 patients in the EL_INST cohort were seizure-free at 12 months postoperatively.

From a total of 19 full-text articles, 23 cases with 25 intra-axial lesions (mean age 36.7 ± 12 years, range 21–67 years, 35% female, mean disease duration 7.4 ± 9 years, range 0.4–27 years) with tumor-related epilepsy were identified (Table 1). A detailed description of these cases is provided in Supplementary Table 1. The DSC index between our EL_INST cohort and EL_LIT was 0 for 11 of 25 lesions and the highest value was 0.02, indicating negligible anatomical overlap between lesions in the two cohorts.

### Descriptive analysis of connectivity hubs potentially implicated in epileptogenicity

**Figure 3:**
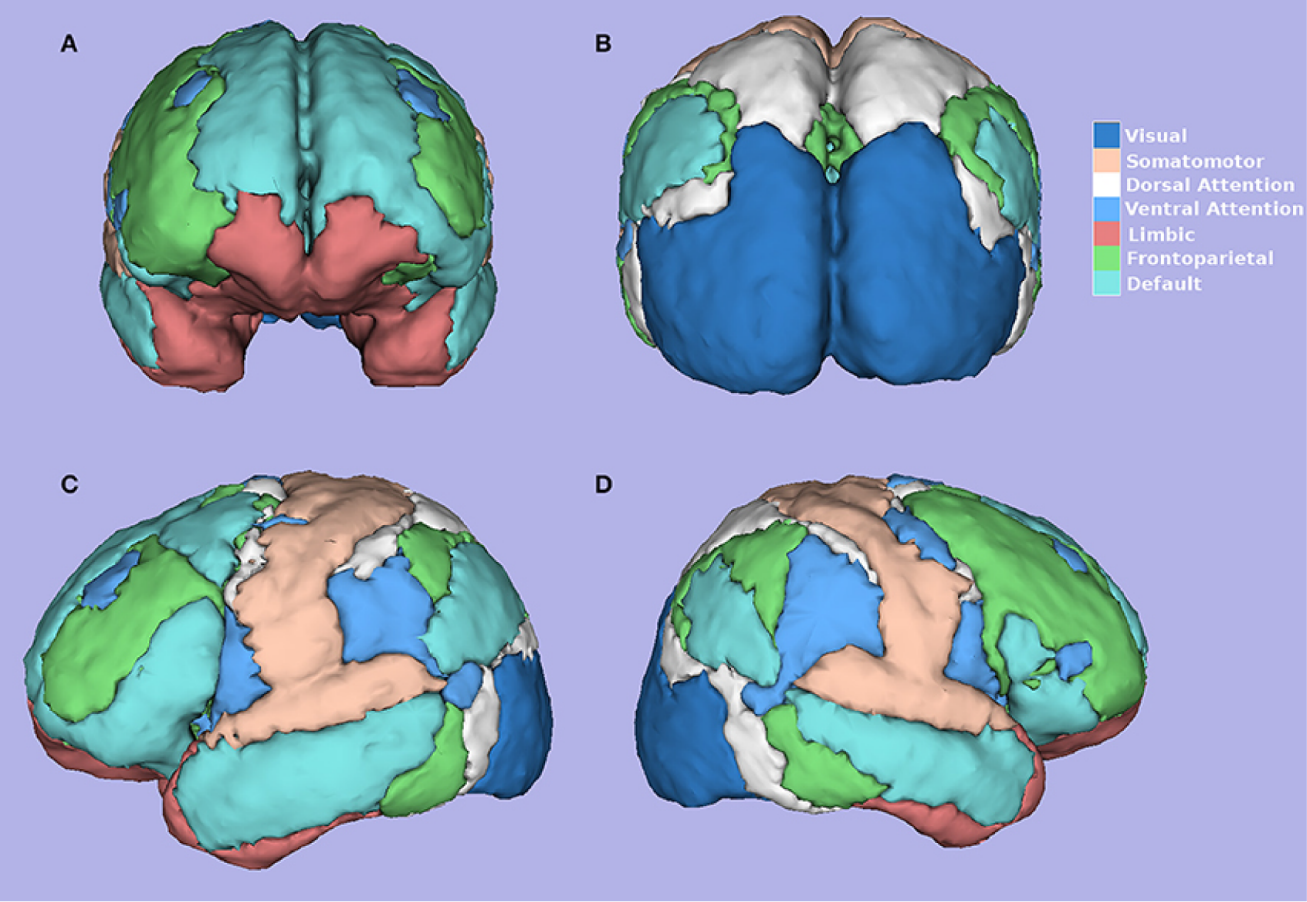
(**A**) Epileptogenic network associated with tumors. Summed maps computed using the binary individual connectivity maps from EL_INST (shaded red) and literature (shaded green) were thresholded at 50% (i.e., at least 50% of lesion connectivity overlap) and shown on T1-weighted (MNI brain) for visualization purposes. (**B**) Graphical presentation of 7 resting state networks. Figure reproduced with permission from Rojas et al.^27^.

Despite the low degree of anatomical overlap between lesions in EL_INST and EL_LIT, we found extensive overlap in connectivity patterns. Calculating the percentage of volume overlap between both 50% thresholded summed connectivity maps (i.e., voxels significantly connected to at least half of the cohort lesions) and anatomical ROIs specified in the AAL atlas, we observed prominent shared connectivity to the middle temporal gyri, dorsal anterior cingulate, posterior cingulate, and pre-cuneus (Fig. 3A). Similarly, the top 4 canonical resting-state networks implicated in epileptogenicity, as determined by the percentage of volume overlap between both 50% thresholded EL summed connectivity maps and the resting-state network labels, were the Default Mode Network (DMN, 19.5%), Frontoparietal Network (19.1%), Ventral Attention Network (19.1%), and the Limbic Network (12.5%). The 7 canonical resting-state networks have been provided as reference in Fig. 3B.

### Exploratory analysis of regions and resting-state networks specifically engaged by epileptogenic lesions, compared to non-epileptogenic lesions

To quantify the odds of particular resting-state networks being involved in epileptogenicity specifically, we conducted a comparison between the EL_INST and NEL_INST cohorts using voxelwise odds-ratio (VOR) maps. The lesions from EL_LIT were excluded from this analysis due to concerns regarding the limitations of combining 2D segmentation approaches with actual 3D techniques possible using our in-house cases.

**Figure 4:**
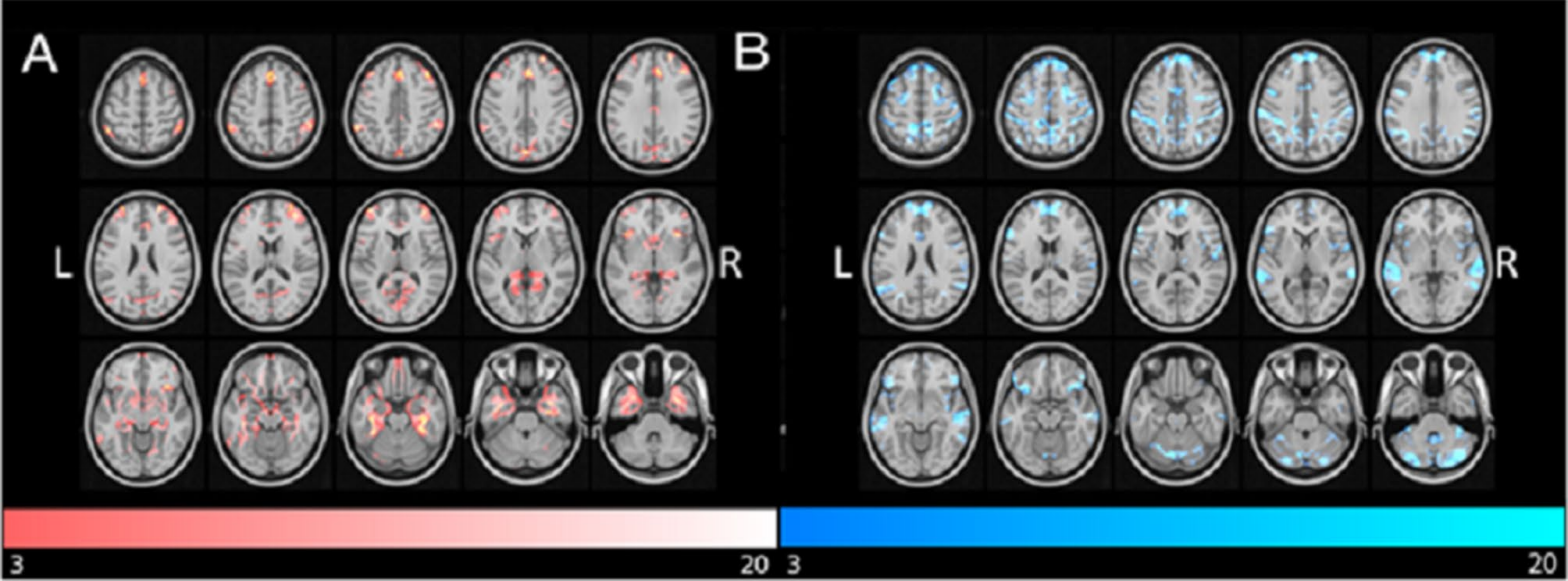
VOR Maps based on EL_INST and NEL_INST. Voxelwise odds ratios of regions most likely connected with (**A**) epileptogenic lesions versus (**B**) non-epileptogenic lesions.

Voxels in the EL_INST and NEL_INST cohorts with peak VORs between 1 and 20 are outlined in Fig. 4 (A and B, respectively). Through this approach, the bilateral medial frontal gyri, right parahippocampal gyrus, right temporal pole, and right inferior parietal lobule constituted the top 5 functionally-connected anatomical locations with EL_INSTs (Supplementary Table 2). Conversely, bilateral cerebella, bilateral precuneus, and right rolandic operculum had the greatest likelihood of functional connectivity with NEL_INSTs (Supplementary Table 3). Because the median volume of EL_INSTs tended to be larger than NEL_INSTs (3,450.5 ± SD versus 737 cc ± SD, *p* > 0.05), we confirmed that the connectivity findings were not due to larger lesions in the EL_INST group. The overall volumes of the binarized thresholded connectivity maps were similar (325.8 cc ± SD versus 294.4 cc ± SD, *p* > 0.05), suggesting that (1) after the thresholding connectivity step, the extent of connectivity is similar across lesions and (2) location—rather than size—is a major driver of the connectivity differences (Table 1).

Given that anatomical regions do not necessarily correlate with function, group differences in functional connectivity to canonical resting-state networks were also examined. Implementing a mean VOR > 1.5 threshold, we identified the Limbic (mean VOR = 1.92) and the Frontoparietal Networks (mean VOR = 2.04) to be most associated with epileptogenicity. These results are summarized in Supplementary Table 4. Conversely, the Dorsal (mean VOR = 2.12) and Ventral (mean VOR = 1.52) Attention Networks along with the DMN (mean = VOR 2.08) were resting-state networks most likely to be functionally connected with NELs (Supplementary Table 5). A graphical representation of the 7 established resting state networks is provided in Fig. 3 for reference^32^. In this approach, it is important to note that although several individual VOR peaks overlapped with regions in the DMN for EL_INSTs, the averaging of the volume of these peaks in the context of the entire DMN ROI volume resulted in smaller mean VOR values.

## Discussion

The LNM method is an emerging neuroscientific method that can facilitate exploration of network connectivity in individuals with neurological symptoms stemming from lesions in seemingly disparate brain regions. The majority of landmark studies using this technique, however, have focused on focal destructive lesions^20–22^. In this proof of concept study, we applied LNM to cases of epilepsy, secondary to intra-axial mass lesions. Despite the absence of significant anatomical overlap between EL_INSTs, we identified discrete functional connections with canonical resting-state networks that have been previously implicated in focal or generalized epilepsy. The connectivity pattern was externally validated with ELs identified from the literature.

### Resting-state networks connected with epileptogenicity

Despite a low degree of anatomical overlap between lesions within EL_INST and EL_LIT, we were able to identify distinct functional connectivity hubs. The percentage volume overlap between these hubs and canonical resting-state networks was highest in the Frontoparietal, Limbic, DMN, and Ventral Attention Networks. Using a cohort of non-epileptogenic lesions (NEL_INST), we were further able to quantitatively explore the differences in the odds of particular resting-state networks being more vulnerable to epileptogenicity. Although our approach was limited by differences in tumor histology, it does highlight the importance of comparing with a “control group”. In our case, the DMN was highly linked with our non-epileptogenic mass lesions but less so in our epileptgenic network, as shown by the VOR analysis. One explanation could be that resting-state networks such as the DMN involve larger brain areas and thus have a higher likelihood of overlapping functional hubs across the brain. Hence the importance of conducting a VOR analysis with a “control” group. Through this approach, we were able to show that while the Limbic and Frontoparietal Networks, both previously described in clinical experience in people with epilepsy^33,34^, continued to show a high likelihood of being functionally connected with EL_INSTs, the Dorsal and Ventral Attention Networks along with the DMN were in fact more likely to be functionally connected with NEL_INSTs. The areas within the Limbic Network play a key role in epilepsy secondary to their ability to produce and propagate synchronized physiological activity^35,36–39^. Furthermore, structural changes in the thalamus (part of the limbic network), including loss of volume over time, have been reported in various forms of epilepsy suggesting the critical role of this structure within a broader epilepsy network^32,40,41^. In addition, the interconnection of limbic structures through thalamic nuclei, particularly the anterior thalamic nucleus, has been a target for stimulation in the management of epilepsy not amenable to curative resection^42^.

Our finding that the DMN had a lower likelihood of functional connectivity with EL_INST, compared with NEL_INST, is also different from what has been shown with mTLE, for which numerous publications on associated networks have suggested the importance of both the DMN and the Limbic Network^18,43–46^. However, we did find an increased likelihood of functional connectivity with the Frontoparietal Network, which has broad connections with various brain networks, serving as a global functional hub^47^. Therefore, we postulate that while the Limbic Network may be a common conduit for seizure propagation in both epilepsy secondary to mass lesions and mTLE, the two forms of epilepsy may differ in their functional association with the DMN and the Frontoparietal Networks. Notably, our observed connectivity pattern was validated in an external cohort of 25 epileptogenic lesions identified from the literature (EL_LIT). This was despite the low anatomical overlap—indicated by the DSC index—between lesions in EL_INST and EL_LIT. It is important to emphasize that our study cannot make any conclusions regarding the strength or direction of connectivity with individual regions within these networks, given our conservative approach using binary of connectivity masks.

### Implications for research and practice

Demonstrating the engagement of Limbic Network by EL_INSTs not only reaffirms the important role of this canonical resting-state network in epilepsy but also provides preliminary evidence for our approach in applying LNM to larger intra-axial mass lesions. Application of this methodology to larger datasets of lesional MRE, including mTLE, can further validate our methods and provide additional insight into the resting-state networks implicated in epileptogenicity. Serving to direct our research focus to distinct resting-state network-based hypotheses, these can subsequently be supplemented with imaging (functional and structural) and neurophysiological methods to better understand the pathophysiology of seizure generation, propagation, and termination.

The underlying pathological diagnosis for MRE subjects is heterogeneous. Accepting that additional microenvironmental and structural factors are involved in the epileptogenicity of certain lesions (e.g., LGGs and cavernomas), LNM can be used to identify locations vulnerable to seizures (due to association with vulnerable networks) and, by extension, may help predict post-surgical seizure outcomes. Recently, a network-targeted approach to the surgical management of epilepsy was implemented, based on preoperative and postoperative native resting state functional imaging; in this study, functional imaging as a biomarker of postoperative seizure freedom had a 93% specificity and 96% sensitivity^48^. Given that many subjects in our study were seizure-free postoperatively, it was not possible to make meaningful statistical predictions regarding connectivity patterns predictive of seizure-freedom. We postulate that preoperative functional imaging can serve as a valuable biomarker in epileptogenic tumors as well; however, functional imaging is currently not routinely performed in neuro-oncology. Validation of the LNM approach may obviate the need for native functional imaging.

### Limitations

Given the novelty of LNM as a research tool in neuroscience and the proof of concept nature of our study, it is important to interpret the results of our study with caution. Although our normative database was based on a large sample of ~ 1,000 individuals^28^, the population is comprised of healthy individuals, not necessarily age-matched with our cohorts. With any neurological disorder, including epilepsy, it is well-known that functional and structural connectivity can be vastly different between pathologic and healthy brains^24^. However, the MRI hardware and acquisition parameters used in constructing normative connectivity datasets are highly optimized, enabling potentially more reliable connectivity patterns with greater reproducibility than native rsfMRI. Furthermore, our conservative strategy of thresholding and binarizing connectivity masks was helpful in identifying courser patterns of connectivity—rather than granular differences in connectivity strength—that are more likely to be preserved in epilepsy patients.

In mTLE, native rsfMRI studies have suggested a difference in connectivity patterns between left- and right-sided pathology^43,49^. Our EL_INST cohort was comprised mostly of left-sided lesions. Furthermore, it would be ideal to establish a link between seizure semiology and connectivity pattern. Our dataset was too small to draw meaningful conclusions regarding lesion laterality and connectivity patterns, but this should be addressed in future studies. Furthermore, validation of our findings with an external cohort of ELs adds credence to our results.

The pathological entities in the EL_INST cohort were heterogeneous, comprised of neoplastic lesions and neuronal/vascular malformations. Each pathology is associated with a different pathophysiology of seizure induction^35^. Furthermore, our NEL_INST cohort was comprised exclusively of brain metastases, lesions that do not typically cause seizures. In addition, the specific interaction of each lesion with nearby brain tissue is distinct and the irritative zone may span beyond the area segmented manually by us as part of the ROI^50^. However, our identification of distinctly shared functional connectivity networks despite this heterogeneity, and our ability to externally validate these findings, further supports the feasibility of LNM in identifying vulnerable resting-state networks that, when exposed to the necessary substrate, can induce seizures/epilepsy.

## Conclusions

In this proof of concept study of LNM, we have reaffirmed that epilepsy secondary to intra-axial mass lesions is a brain network-wide phenomenon. We have shown the potential involvement of canonical resting-state networks and suggested the possibly higher vulnerability of the Limbic and Frontoparietal Networks toward epileptogenicity. Most importantly, we have demonstrated that LNM can be feasibly applied to large mass lesions. Given the methodological limitations of LNM and the preliminary nature of our study, despite our validation with an independent dataset from the literature, larger prospective studies with age- and pathology-matched controls are needed to validate this approach prior to its broader scale application in epilepsy and large mass lesions. LNM could potentially obviate the need for DTI and fMRI, providing significant cost and practical advantages to the clinical and research workflow.

## Supplementary information

Supplementary file1 (PDF 56 kb)
