# Supplementary material for: Identification of neural networks preferentially engaged by epileptogenic mass lesions through lesion network mapping analysis
Source: Sci Rep. 2020 Jul 3;10:10989. doi: 10.1038/s41598-020-67626-x (PMC7335039; doi:10.1038/s41598-020-67626-x)
Supplement: Supplementary file 1 — Supplementary file1 (PDF 56 kb) [file 41598_2020_67626_MOESM1_ESM.pdf]

Identification of neural networks preferentially engaged by epileptogenic mass lesions: A lesion network mapping analysis

Authors:

Alireza Mansouri MD MSc[1*]
Jürgen Germann, M.Sc.[2]
Alexandre Boutet, M.D.[2, 3]
Gavin J. B. Elias, B.A.[2]
Karim Mithani BSc MEng [4]
Clement T. Chow BKin[2]
Brij Karmur BSc[4]
George M. Ibrahim MD PhD[5,6,7]
Mary Pat McAndrews PhD [8]
Andres Lozano MD PhD[9]
Gelareh Zadeh MD PhD [9]
Taufik A. Valiante MD PhD [9]

[*]Corresponding author: Alireza Mansouri / amansouri@pennstatehealth.psu.edu

[1] Department of Neurosurgery, Penn State Health, Hershey, PA
[2] University Health Network, Toronto, ON
[3] Joint Department of Medical Imaging, University of Toronto, Toronto, ON
[4] Faculty of Medicine, University of Toronto, Toronto, ON
[5] Associate Scientist, Program in Neuroscience and Mental Health, Sickkids Research Institute, Toronto, ON
[6] Division of Neurosurgery, The Hospital for Sick Children, Toronto, ON
[7] Institute of Biomaterials and Biomedical Engineering, Department of Surgery, University of Toronto, Toronto, ON
[8] Department of Neuropsychology, University Health Network, Toronto, ON
[9] Division of Neurosurgery, University Health Network, Toronto, ON

Supplementary Table 1. Clinical and tumor characteristics of lesions identified from the literature for external validation

Author (year)	Age (years)	Sex	Tumor Type	Grade	Impacted structure	Seizure Type	Seizure Duration (years)
Aaron (1984)	21	M	Neoplasm	NA	Right temporal lobe	Partial, complex	20
	45	M	Astrocytoma	3	Left temporal lobe	Generalized	0.25
Alimohamadi (2016)	30	F	Glioma	2	Left temporal lobe	NA	0.33
Anneken (2006)	48	F	Astrocytoma	2	Left frontotemporal lobe	Generalized tonic-clonic	7
Bai (2015)	26	F	Astrocytoma	2	Left inferior temporal lobe	Complex partial	0.04
Buklina (2014)	23	M	Astrocytoma (anaplastic)	3	Left inferior precentral gyrus	Clonic seizure (tongue)	0.66
Buklina (2014)	52	M	Astrocytoma	2	Left superior & middle temporal gyri	Generalized	12
Chowdhury (2010)	41	M	Ganglioma (anaplastic)	3	Right amygdala	NA	NA
Duffau (2006)	38	F	Oligoastrocytoma	2	Right insula, temporal pole, mesiotemporal lobe	Generalized	4
Duffau	39	M	Glioma	2	Right frontal pole	NA	0.66

(2016)	41	F	Glioma	2	Right frontal pole	NA	0.66
Garcia Pulido (2013)	38	F	Oligodendroglioma (multicentric)	2	Left frontal, parietal, occipital lobes	Generalised tonic-clonic	27
Harmsen (2019)	25	M	Glioma (angiocentric)	1	Right frontal lobe	NA	2
Henry (1994)	24	F	Astrocytoma	2	Right frontal, inferior temporal lobes, insula	Complex partial	16
Hoque (2009)	67	M	Astrocytoma	3	Left lateral perirolandic cortex	Simple partial, clonic (tongue)	NA
Kennedy (2013)	38	M	Oligodendroglioma	2	Right temporal lobe	NA	NA
Maesawa (2016)	24	M	Pleomorphic xanthoastrocytoma	2	Left deep parietal operculum, transverse parietal gyrus	Medically refractory daily	7
Majores (2008)	45	M	Ganglioma (anaplastic)	3	Left temporo-mesial lobe	Single, generalized	NA
	60	M	Ganglioma (atypical)	2	Right frontocentral lobe	Refractory epilepsy	NA
	37	M	Ganglioma (atypical)	2	Left frontal lobe, insula, basal ganglia	Single, generalized	NA
Marucci	27	M	Intracerebral hamartoma	NA	Left frontal lobe	Tonic seizure	20

(2011)						(oculo-cephalic)	
Vajkoczy (1998)	27	NA	Astrocytoma	2	Left temporo-mesial lobe	Intractable complex partial	9.6 (mean)
Whittle (1992)	28	F	Astrocytoma (anaplastic)	3	Right mid-rolandic cortex	Partial Left sensory	0.66

Supplementary Table 2. Anatomical areas with the greatest likelihood of being functionally connected with epileptogenic versus non-epileptogenic lesions (AAL Atlas)

Brain Region	Mean Voxelwise Odds Ratio
**Right Medial Frontal Gyrus**	4.227846
**Left Medial Frontal Gyrus**	4.010104
**Right Parahippocampal Gyrus**	3.993287
**Right Temporal Pole**	3.828816
**Right Inferior Parietal Lobe**	3.745296
**Right Middle Frontal Gyrus**	3.736978
**Left Parahippocampal Gyrus**	3.696352
**Left Temporal Pole**	3.64159
**Left Fusiform Gyrus**	3.469102
**Right Anterior Cingulate Cortex**	3.426886
**Right Superior Frontal Gyrus**	3.244597
**Left Inferior Temporal Lobe**	3.230719
**Right Fusiform Gyrus**	3.176906
**Left Calcarine Fissure**	3.140045
**Left Cuneus**	3.127826
**Left Caudate Nucleus**	3.124714
**Right Supramarginal Gyrus**	3.102246
**Left Inferior Parietal Lobe**	3.086116
**Left Globus Pallidus**	3.064151

Supplementary Table 3. Anatomical areas with the greatest likelihood of being functionally connected with non-epileptogenic versus epileptogenic lesions (AAL Atlas)

Brain Region	Mean Voxelwise Odds Ratio
**Right Cerebellum**	9.581622
**Left Cerebellum**	5.37431
**Right Precuneus**	2.988474
**Left Precuneus**	2.922513
**Right Rolandic Operculum**	2.831945
**Left Anterior Cingulate Cortex**	2.620148
**Right Precentral Gyrus**	2.404755
**Left Precentral Gyrus**	2.349517
**Left Rolandic Operculum**	2.111296

Supplementary Table 4. Resting-state networks with the greatest likelihood of being functionally connected with epileptogenic versus non-epileptogenic lesions[31]

Functional Network	Mean Voxelwise Odds Ratio
**Limbic Network**	1.92
**Frontoparietal Network**	2.04

Supplementary Table 5. Resting-state networks with the greatest likelihood of being functionally connected with non-epileptogenic versus epileptogenic lesions (Rojas et al.)[31]

Functional Network	Mean Voxelwise Odds Ratio
Dorsal Attention	2.12
Ventral Attention	1.52
Default	2.08
